# Supplementary material for: Predicting mosquito infection from Plasmodium falciparum gametocyte density and estimating the reservoir of infection
Source: eLife. 2013 May 21;2:e00626. doi: 10.7554/eLife.00626 (PMC3660740; doi:10.7554/eLife.00626)
Supplement: Figure 1—source data 1. — DOI: http://dx.doi.org/10.7554/eLife.00626.004 [file elife00626s001.docx]

| **Country** | **Burkina Faso (Ouédraogo et al., 2009)** | **Kenya (Schneider et al., 2007)** |
| --- | --- | --- |
| Numbers of hosts | 71 | 100 |
| Mean no. of assays per blood sample (range) | 2.63 (2, 4) | 1.00 (1) |
| Mean no. of mosquitoes dissected per host (range) | 28.9 (4, 53) | 30 (30) |
| Mean % mosquitoes infected (range) | 5.43 (0, 56) | 4.57 (0, 33) |
| Mean no. asexual parasites µl^-1^ (range) | 3 418 (0, 58 751) | 308 (0, 19 520) |
| Mean age of host in yr (range) | 7.09 (2.91, 13.6) | 5.03 (1.15, 10.9) |
| Method of host selection | Random | Uncomplicated malaria cases |
| Prior treatment with drugs* | No | Yes |

*Patients were treated with either Artemisinin Combination Therapy or Sulphadoxine-Pyrimethamine 14 days prior to sample collection
