# Supplementary material for: Predicting mosquito infection from Plasmodium falciparum gametocyte density and estimating the reservoir of infection
Source: eLife. 2013 May 21;2:e00626. doi: 10.7554/eLife.00626 (PMC3660740; doi:10.7554/eLife.00626)
Supplement: Figure 1—source data 2. — Lower DIC values indicate a more parsimonious fit to the data. All models were fitted with gametocyte density on the arithmetic scale. There was no evidence of any difference between study sites (DIC of best-fit model allowing mosquito infection to vary between study location = 1042). The best-fit model on the logarithmic scale was the Gompertz model (DIC = 1053). DOI: http://dx.doi.org/10.7554/eLife.00626.005 [file elife00626s002.docx]

| **Function name** | **DIC value** | **Best fit parameters**  **(95% Bayesian credible intervals)** | | | | | | | |
| --- | --- | --- | --- | --- | --- | --- | --- | --- | --- |
|  |  | $\beta_{0}$ | $\beta_{1}$ | $\sigma$ | $\eta$ | $z_{1}=1$ | $z_{1}=2$ | $z_{2}=1$^¤^ | Shape parameters |
| Constant | 1111 | -2.74  (-3.3, -2.2) | 77.2  (65, 90) | 7.17  (6.0, 9.0) | 0.0422  (8.8x10^-3^, 0.25) | -0.404  (-0.62, -0.10) | 0.579  (0.18, 1.1) | 0.484  (0.15, 0.92) | $\alpha_{0}$=0.0535 (0.043, 0.066) |
| Linear | 1073 | -3.22  (-3.8, -2.6) | 78.5  (57,90) | 7.93  (6.5, 9.8) | 0.0487  (9.6x10^-3^, 0.31) | -0.387  (-0.048, -0.61) | 0.411  (-0.012, 0.95) | 0.0888  (-0.17, 0.43) | $\alpha_{0}$=0.0429 (0.032, 0.059)  $\alpha_{1}$=2.31x10^-4^ (9.5x10^-5^, 6.0 x10^-4^) |
| Power | 1059 | -2.89  (-3.4, -2.3) | 78.1  (61, 89) | 7.80  (6.5, 9.6) | 0.0176  (1.2x10^-5^, 0.094) | -0.474  (-0.21, -0.66) | 0.256  (1.6x10^-3^ , 0.56) | 0.102  (-0.05, 0.34) | $\alpha_{0}$=0.0341 (0.014, 0.053)  $\alpha_{1}$=4.09x10^-3^ (4.5x10^-4^, 0.018)  $\alpha_{2}$=0.521 (0.30, 0.78) |
| Hyperbolic | 1062 | -3.03  (-3.6, -2.3) | 78.9  (62, 82) | 7.99  (6.6, 9.9) | 0.0591  (6.3x10^-3^, 0.23) | -0.396  (-0.055, -0.62) | 0.402  (-0.015, 0.99) | 0.0901  (-0.17, 0.44) | $\alpha_{0}$=0.0333 (0.023, 0.046)  $\alpha_{1}$=2.86x10^-4^ (7.5x10^-5^, 8.2 x10^-4^)  $\alpha_{3}$=5.03 (2.1, 8.3) |
| Sigmoid | 1038 | -3.05  (-3.4,-2.4) | 78.3  (67,89) | 8.13  (6.8, 9.8) | 0.0495  (0.021, 0.20) | -0.226  (-0.53, 0.21) | 0.872  (0.34, 1.5) | 0.127  (-0.24, 0.59) | $\alpha_{0}$=0.0370 (0.029, 0.042)  $\alpha_{1}$=0.130 (0.089, 0.19)  $\alpha_{2}$=18.2 (8.3, 73)  $\alpha_{3}$=0.0124 (0.023, 0.026) |
| Gompertz | 1034* | -2.88  (-3.8, -2.3) | 78.0  (66, 89) | 7.87  (6.6, 9.6) | 0.0446  (9.1x10^-3^, 0.14) | -0.181  (-0.51, 0.14) | 0.881  (0.39, 1.4) | 0.0904  (-0.28, 0.44) | $\gamma_{0}$=0.0382 (0.029, 0.049)  $\gamma_{1}$=0.165 (0.093, 0.31)  $\gamma_{2}$=51.4 (5.7, 1037)  $\gamma_{3}$=0.0129 (3.0x10^-3^, 0.044) |

*best fit model ^¤^positive values indicate greater infectivity in older age group
