# Supplementary material for: Predicting mosquito infection from Plasmodium falciparum gametocyte density and estimating the reservoir of infection
Source: eLife. 2013 May 21;2:e00626. doi: 10.7554/eLife.00626 (PMC3660740; doi:10.7554/eLife.00626)
Supplement: Figure 2—source data 1. — Asexual parasite density was estimated by microscopy while gametocyte density was estimated using QT-NASBA. DOI: http://dx.doi.org/10.7554/eLife.00626.007 [file elife00626s003.docx]

| **Age group (yr)** | **Number of hosts** | **Mean age (yr)** | **Mean asexual parasite density µl^-1^ (range)** | **Mean gametocyte density µl^-1^**  **(range)** |
| --- | --- | --- | --- | --- |
| [ 0–5 ) | 79 | 3.9 | 5357 (0, 50 112) | 83.6 (0, 1 402) |
| [ 5–10 ) | 97 | 7.2 | 3094 (0, 63 068) | 174.3 (0, 5 985) |
| [10–15 ) | 78 | 12.2 | 838 (0, 5 088) | 47.2 (0, 502) |
| [15–20 ) | 40 | 16.9 | 465 (0, 4 592) | 43.4 (0, 419) |
| [20–30) | 41 | 24.0 | 706 (0, 9 694) | 64.9 (0, 910) |
| [30–40) | 29 | 35.2 | 314 (0, 5 662) | 46.1 (0, 684) |
| [40–50) | 21 | 45.7 | 1607 (0, 24 272) | 13.7 (0, 97) |
| [50+ | 27 | 59.5 | 579 (0, 14 636) | 11.2 (0, 84) |
