# Supplementary material for: Predicting mosquito infection from Plasmodium falciparum gametocyte density and estimating the reservoir of infection
Source: eLife. 2013 May 21;2:e00626. doi: 10.7554/eLife.00626 (PMC3660740; doi:10.7554/eLife.00626)
Supplement: Figure 2—source data 2. — DOI: http://dx.doi.org/10.7554/eLife.00626.008 [file elife00626s004.docx]

| **Name** | **Description** | **Best fit parameters**  **(95% credible interval)** | | | **DIC value** |
| --- | --- | --- | --- | --- | --- |
|  |  | $\tau$ | $\psi$ | $\omega$ |  |
| Gametocyte age profile | How the mean number of gametocytes ml^-1^ blood changes with host age | 0  (0, 0) | 22.7  (17, 32) | 0.0934  (0.080, 0.11) | 1532 |
| Reservoir of infection age profile | How the probability that a blood-feeding mosquito will develop oocysts changes with host age | 0.0410  (0.024, 0.11) | 0.305  (0.19, 0.63) | 0.0298  (0.017, 0.041) | 1179 |
